# Laypeople and dental professionals' perception of the aesthetic outcome of two treatments for missing lateral incisors

**DOI:** 10.1002/cre2.504

**Published:** 2021-10-15

**Authors:** Cecilia Hedmo, Rune Lindsten, Eva Josefsson

**Affiliations:** ^1^ Department of Orthodontics The Institute for Postgraduate Dental Education Jönköping Sweden; ^2^ School of Health and Welfare Jönköping University Jönköping Sweden

**Keywords:** aesthetic outcome, aplasia, implants, maxillary laterals, orthodontic space closure, orthodontics

## Abstract

**Objective:**

To investigate laypeople and dental professionals' opinions of the aesthetic outcome from implant therapy (IT) and orthodontic space closure (SC) for missing maxillary lateral incisors.

**Material and methods:**

Evaluation was performed by three groups: laypeople 20–30 years of age (*n* = 26), laypeople 50–70 years of age (*n* = 26) and orthodontists (*n* = 25). The assessors viewed photographs of 44 different cases treated with IT or SC, and made an evaluation of the aesthetics.

**Results:**

The gingival color adjacent to the replaced tooth was rated as having better aesthetics in the SC cases (*p* = 0.000). The orthodontists preferred the aesthetics of the dentition in the SC cases (*p* = 0.042). The young laypeople, compared to the older laypeople and orthodontists, were more dissatisfied with the color of the tooth replacing the missing lateral incisor in SC cases (*p* = 0.043).

**Conclusion:**

The color of the gingiva adjacent to the implant‐supported crowns had a lower aesthetic rating than the SC group. Laypeople rated both treatments as equally good. The orthodontists had a slight preference for the aesthetics in the SC cases.

## 1 INTRODUCTION

Missing maxillary lateral incisors is a common feature in the orthodontic diagnosis panorama (Krassnig & Fickl, 2011). Agenesis of one or more teeth is a consequence of a disruption in the initiation of the formation of the tooth (Proffit et al., 2013). The teeth most often affected are the mandibular and maxillary second premolars and the lateral incisors in the maxilla (Thilander & Myrberg, 1973). The prevalence of agenesis of the maxillary lateral incisor varies between 1.5% and 2.0%, which has been reported in several studies (Grahnén, 1956; Polder et al., 2004; Robertsson & Mohlin, 2000).

Available space, age of the patient, type of malocclusion, status of adjacent teeth, facial profile, lip line and gingival contour are factors that should be taken into account when choosing treatment in cases with missing maxillary lateral incisors (Kokich, 2001; Zachrisson, 2007).

There are three major types of treatment options: space closure (SC), implant‐supported crowns or tooth‐supported fixed prostheses. There are no differences in the duration of the treatment when comparing SC or orthodontic redistribution of space in the dental arch for prosthetic replacements (Seehra et al., 2020), thus the clinician should choose the least invasive treatment with the best long‐term prognosis concerning aesthetics and function. In many patients, there are several treatment options possible.

Osseo‐integrated implants are reported to be the most commonly used therapy when a missing maxillary lateral incisor needs to be substituted (Kinzer & Kokich, 2005; Priest, 2019) but the use of temporary anchorage devices have increased the possibilities for orthodontic treatment only (Amm et al., 2019). Some cases require space opening when implant therapy (IT) is planned, but pre‐prosthetic orthodontic treatment is recommended to be delayed for as long as possible (Beyer et al., 2007; Uribe et al., 2013). This is because atrophy of the alveolar ridge can occur if space opening is performed too early. Some unwanted complications when replacing missing teeth with implant supported crowns have been found, including the degeneration of the soft tissue, discolored gingiva, visible implants, and infra‐occlusion of the implant‐supported crown (Thilander et al., 2001). Different anatomical conditions also influence the aesthetic prognosis, such as bone volume, size of the papilla, and arch form. Björk and Skieller (1983) have shown that craniofacial skeletal alterations continue even during adulthood, and Iseri and Solow (1996) found a continuing eruption of the maxillary incisors and first premolars up to the age of 25. These findings have raised many questions about the long‐term result of IT in the aesthetic zone and whether this is a good treatment alternative in the young patient.

Perception of dental aesthetics can vary between laypeople and professionals (Armbruster et al., 2004a; Schneider et al., 2016) and can also vary between different treatments (Qadri et al., 2016). Laypeople's opinions are of great interest since they are potential critics. Evaluations of aesthetical outcome in patients treated due to missing maxillary lateral incisors have been performed (Armbruster et al., 2004a; De‐Marchi et al., 2014; Schneider et al., 2016). In a subsidized system, and where one strives to provide minimal invasive dentistry and yet aesthetics to fulfill the patients expectations, the results can differ. When aesthetics are evaluated there is a risk that only the better cases are selected and presented in the literature, therefore it is of interest to have several comparative studies performed.

The aim of this study was to examine laypeople and dental professionals' opinions of the aesthetic outcome from IT and orthodontic SC for missing maxillary lateral incisors.

## 2 MATERIAL AND METHODS

This study was approved by the Research Ethics Committee, Faculty of Health Sciences, Linköping University, diary number 2013/428‐31. All participants in this study, both patients representing the cases to be assessed and the assessors, gave written consent to participate in this study.

A retrospective cross‐sectional, quantitative study design was used. Photographs of patients who had one or two missing maxillary lateral incisors and had been treated with either orthodontic SC or IT were evaluated by laypeople and orthodontists. Extra‐oral photographs of the lower face and intraoral photographs of teeth and gingiva were used. The photographs were taken by one of the authors (E. J.) using the same digital camera in a predetermined sequence, lighting, and position. The images were not manipulated. The photographs were presented to the assessors printed on paper to ensure that the colors were similar to all the assessors.

The subjects were recruited at the Institute for Postgraduate Dental Education, Jönköping, Sweden, all patients who had undergone IT due to missing one or two maxillary lateral incisors and treated between 2001 and 2008 were asked to participate in the present study. This group was matched with a group treated with orthodontic SC, with respect to diagnosis and sex. Patients with cleft lip and palate or oligodontia were excluded.

The implant installations were performed before the patients were 25 years old, between the years 2001 and 2008. The surgical procedure was performed at the Departments of Oral and Maxillofacial Surgery or Periodontology by eight different specialists and the prosthetic treatment was performed by seven different specialists in prosthodontics. Forty‐one patients with IT met these criteria, 20 males and 21 females. The photographs were taken at least 5 years after the prosthetic therapy and the patients were 24 years and 7 months to 33 years and 8 months old.

The patients were contacted by letter and were informed about the study. Twenty‐two of these subjects agreed to participate in the study, 8 males and 14 females born between 1979 and 1989.

The group treated with SC consisted of 22 patients, born between 1984 and 1994, registered at the Department of Orthodontics between 2002 and 2004. They were matched to the implant group according to diagnosis, sex and number. The treatment planning and the treatments were performed at the Department of Orthodontics by 11 different orthodontists. The patients in this group (SC) had been treated with fixed orthodontic appliances. These patients were contacted by letter and informed about the study; all of them agreed to participate. The photographs were taken at least 5 years after the orthodontic treatment was finished, at ages between 20 years and 6 months and 30 years and 8 months.

In the IT group there were 7 cases with bilateral aplasia of the maxillary lateral incisor and 15 cases with unilateral aplasia. In the SC group there were 12 patients with bilateral aplasia of the maxillary lateral incisor and 10 patients with unilateral aplasia.

All patients who agreed to participate in this study were included in the assessments. No case was excluded due to poor aesthetic outcome, in order to mirror a standard treatment result in a society with subsidized orthodontic treatment.

Twenty‐five orthodontists in the south of Sweden were asked to participate in the aesthetic evaluation of the photographs. One hundred and fifty‐five randomly selected laypersons, 20–30 years of age, were asked to participate in the aesthetic evaluation, 30 of these agreed to participate and we received answers from 26 of them. Furthermore, 75 randomly selected laypersons, 50–70 years of age, were asked to participate in the evaluation, 28 of these agreed to participate and answers were received from 26 of them. The sample of laypeople was randomly selected from the population and selected from the Swedish Central Population Registry at Statistics Sweden.

Before the assessors were presented with the different cases, they were informed that the pictures they were to assess were of patients with one or two missing anterior teeth in the maxilla and that the patients had been treated with prosthodontics or SC. They were not informed which case had been treated with which treatment method.

The photographs of the cases were printed and were sent to the laypeople and orthodontists for them to assess at home. The tooth or teeth to be assessed were clearly marked. The photographs were in no special order and the photographs of each case were to be assessed on a questionnaire consisting of seven questions, see Supporting Information. Six of these questions were to be answered on a horizontal visual analogue scale (VAS; 100) with the end phrases on the left side: “Very dissatisfied,” and on the right side: “Very satisfied.” The seventh question was a multiple‐choice question and was used to confirm the answer for question number one, the evaluation of the aesthetics in the overall dentition. The answers to questions number one and seven should coincide, otherwise the assessment was excluded.

### 2.1 Statistical method

All statistical analyses were carried out with the statistical software SPSS version 22 (IBM Corp., Armonk, NY, United States). The sample size regarding the number of cases was calculated based on the possibility of detecting a difference of 35% in rating outcome of crown and gingival appearance, with a significance level of 0.05 and power of 80%. The power analysis showed that 22 cases in each group were sufficient. The data was not normally distributed so an independent‐samples Kruskal–Wallis test was used for the statistical analyses between the groups of assessors and a Mann–Whitney *U* test was used for the statistical analyses within the groups of assessors but between treatments. Bonferroni correction was applied for multiple tests. Pearson's correlation test was used to analyze correlations of questions number one and seven within the assessors for each case. To be able to detect a 10‐point deviation between rating groups with power of 0.8 and alpha 0.05, 23 individuals were needed in each group of assessors based on the variation in aesthetic evaluation using VAS (Bowman & Johnston, 2000).

## 3 RESULTS

In total, 77 responses with evaluations of the 44 cases were received. Twenty‐six of them were from the younger laypeople, 26 were from the older laypeople, and the remaining 25 were from the orthodontists. The response rate, out of those who agreed to participate, was 86.7%, for the young laypeople, 92.6% for the older laypeople, and 100% for the orthodontists.

**Figure 1:**
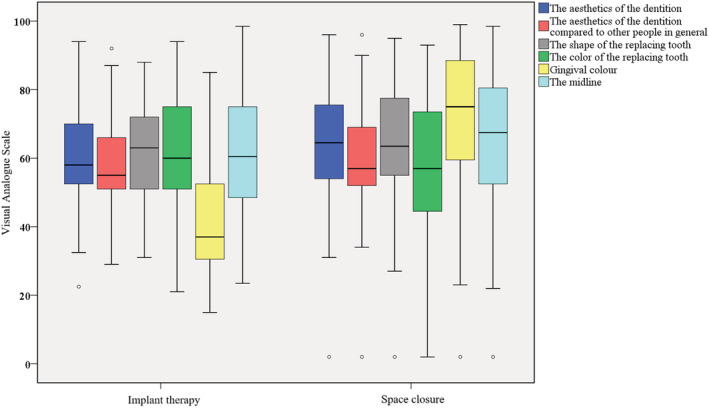
Ratings of implant therapy versus space closure, all assessors merged. Comparing cases with implant‐supported crowns and cases with space closure. The results for each question from the questionnaire are shown in individual boxes and divided between the two different treatment alternatives. Outliers are presented as dots

When comparing cases with implant‐supported crowns and cases with SC, of all the variables rated, there was only a difference concerning the gingival color adjacent to the tooth replacing the missing maxillary lateral incisor, which was rated as having better aesthetics in the space‐closing cases (*p* = 0.000) (Figure 1).

There was no difference in the ratings of the midline between the IT and the SC groups. The orthodontists preferred the aesthetics of the dentition as a whole in the SC cases compared to the cases treated with IT (*p* = 0.042), Table 1. There was no difference between the three rating groups concerning the evaluation of the cases with implant‐supported crowns, except for assessing the midline. The orthodontists rated the midline as better than the young laypeople (*p* = 0.012), Table 2.

**Table 1:** Differences in ratings between the two treatment alternatives within each group of assessors

	Space closure		Implant therapy		
	MED.	CI 95%	MED.	CI 95%	*p*
1. The aesthetics of the dentition					
Orthodontists	69	65–77	62	56–69	0.042^*^
Young laypeople	58	48–76	55	49–66	0.660
Older laypeople	61	55–72	61	53–66	0.694
2. The aesthetics of the dentition compared to other people in general					
Orthodontists	63	55–71	57	53–62	0.294
Young laypeople	54	49–62	53	48–62	0.819
Older laypeople	59	54–68	59	57–67	0.805
3. The shape of the replacing tooth					
Orthodontists	66	58–78	63	57–71	0.567
Young laypeople	62	53–73	57	49–70	0.437
Older laypeople	66	61–79	62	55–70	0.602
4. The color of the replacing tooth					
Orthodontists	63	52–80	62	52–73	0.655
Young laypeople	47	41–61	62	50–72	0.080
Older laypeople	59	53–70	64	53–74	0.332
5. Color of the gingiva adjacent to the replacing tooth					
Orthodontists	89	88–92	37	31–47	0.000^**^
Young laypeople	63	55–77	38	29–44	0.000^**^
Older laypeople	70	62–81	42	33–53	0.000^**^
6. The midline					
Orthodontists	82	72–89	76	56–86	0.052
Young laypeople	53	48–71	53	45–63	0.862
Older laypeople	65	57–73	59	55–68	0.400

*Note*: The distribution of the median (MED.) of the evaluations on the different questions to be answered on a visual analogue scale (VAS; 100). Comparisons between space closure (SC) and implant therapy (IT) within the groups of assessors.

^**^ *p* < 0.001.

^*^ *p* < 0.05.

**Table 2:** Comparisons of ratings between the groups of assessors for the cases treated with implant therapy

	Young laypeople		Older laypeople		Orthodontists		
	MED.	CI 95%	MED.	CI 95%	MED.	CI 95%	*p*
1. The aesthetics of the dentition	54	49–66	60	53–66	59	56–69	0.434
2. The aesthetics of the dentition compared to other people in general	51	48–62	57	57–67	58	53–62	0.120
3. The shape of the replacing tooth	60	49–70	64	55–70	64	57–71	0.461
4. The color of the replacing tooth	50	50–72	64	53–74	58	52–73	0.682
5. The color of the gingiva adjacent to the replacing tooth	39	29–44	42	33–53	36	31–47	0.543
6. The midline	58	45–63	60	55–68	77	56–86	0.012^*^

*Note*: The distributions of the median (MED.) of the evaluations on the questions to be answered on a visual analogue scale (VAS; 100). Comparison between the groups of assessors for each question for the cases treated with implant therapy. Bonferroni correction was applied for multiple tests.

^*^ *p* < 0.05.

Comparing the three groups when evaluating the cases with SC, the younger laypeople were more dissatisfied with the color of the tooth replacing the missing maxillary lateral incisor than the orthodontists (*p* = 0.043), Table 3.

**Table 3:** Comparisons of ratings between the groups of assessors for the cases treated with orthodontic space closure

	Young laypeople		Older laypeople		Orthodontists		
	MED.	CI 95%	MED.	CI 95%	MED.	CI 95%	*p*
1. The aesthetics of the dentition	59	48–76	61	55–72	69	65–77	0.090
2. The aesthetics of the dentition compared to other people in general	56	49–62	57	54–68	65	55–71	0.086
3. The shape of the replacing tooth	63	53–73	65	61–69	63	58–78	0.639
4. The color of the replacing tooth	46	41–61	61	53–70	61	52–80	0.043^*^
5. The color of the gingiva adjacent to the replacing tooth	61	55–77	66	62–81	91	88–92	0.000^**^
6. The midline	65	48–71	67	57–73	84	72–89	0.000^**^

*Note*: The distributions of the median (MED.) of the evaluations on the questions to be answered on a visual analogue scale (VAS; 100). Comparison between the groups of assessors for each question for the cases treated with space closure. Bonferroni correction was applied for multiple tests.

^**^ *p* < 0.001.

^*^ *p* < 0.05.

There was also a difference between the three groups of assessors when assessing the color of the gingiva in the SC cases. The orthodontists were more satisfied with the color of the gingiva adjacent to the replacing tooth compared to the laypeople (*p* = 0.000) and they were more satisfied with the midline compared to the laypeople (*p* = 0.000), Table 3.

The seventh question on the questionnaire was a multiple‐choice question meant to validate the answer to question number one, thus verifying the intra‐examiner reliability within the assessors. When comparing all the assessors, the correlation was *r* = 0.868, *p* < 0.01. The correlation differed between the groups of assessors. For the young laypeople, the correlation was 0.909, for the older ones it was 0.838 and for the orthodontists 0.850, according to Pearson's correlation test (*p* < 0.01). No assessments were excluded due to inadequate consistency.

**Figure 2:**
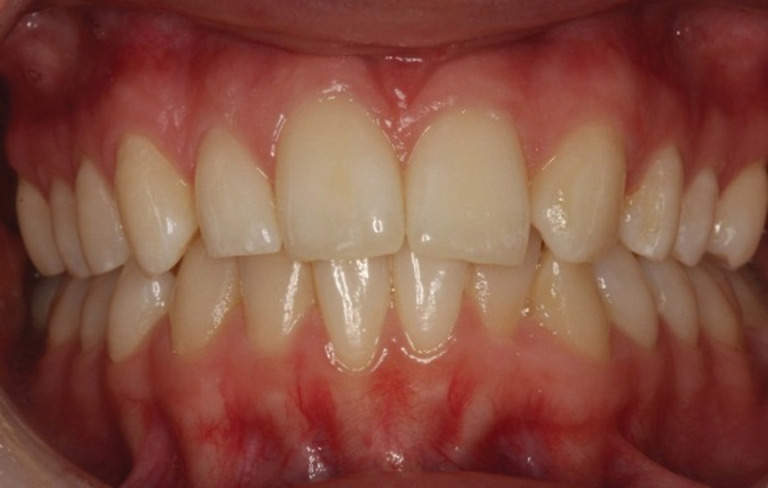
The case rated as the one with the best overall aesthetics of the ones treated with orthodontic space closure

**Figure 3:**
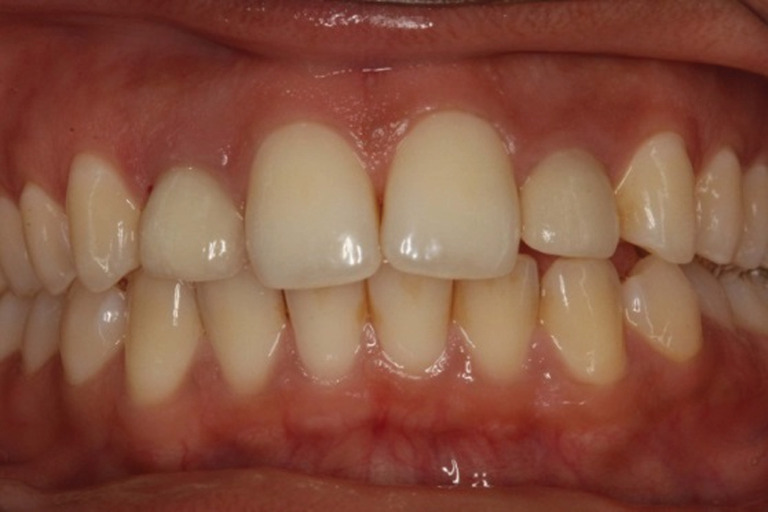
The case rated as the one with the best overall aesthetics of the ones treated with implant supported crowns

The case rated with the best dental aesthetics among the cases treated with SC was a case with a unilateral agenesis of the maxillary lateral incisor and no restorations or whitening had been performed owing to the space‐closing therapy (Figure 2). Among the cases treated with implant‐supported crowns, the case rated with the best overall aesthetics was a case with bilateral agenesis of the maxillary lateral incisors (Figure 3).

## 4 DISCUSSION

Three groups of people evaluated photographs of 44 cases with aplasia of one or both maxillary lateral incisors, either replaced by an implant‐supported crown or by SC. The cases were treated at least 5 years before evaluation. The three groups of assessors consisted of two age groups of laypeople and one group of orthodontists. The number of assessors in each group was judged to be sufficient to give a reliable result based on the findings by Kiekens et al. (2007), who found that a panel of seven randomly selected laypeople or orthodontists is sufficient when assessing the aesthetics in the dentition using photographs and VAS. Visual analogue scales are validated, reliable and widely used when assessing aesthetics (Krishnan et al., 2008; Van Laerhoven et al., 2004).

We found that the color of the gingiva adjacent to the implants was rated lower than the color of the gingiva adjacent to natural teeth. This finding is in agreement of Batra et al. who found that a pigmentation of the gingiva is considered to have a negative impact on the aesthetics according to laypeople (Batra et al., 2018). The color of the replacing tooth is also of concern, especially for young people who rated the color in the SC group slightly lower.

The overall ratings shows no major differences between these two methods to treat patients with missing maxillary lateral incisors, which is in disagreement with Armbruster et al. (2004a) and Qadri et al. (2016), who found that laypeople preferred the aesthetics in patients treated with orthodontic SC compared to patients treated with prosthetic replacement. However, Qadri et al. also found that the age of the assessor had no effect on the aesthetic judgment, which is in agreement with our finding concerning the overall aesthetics.

An asymmetry in the dental arch is more conspicuous than a general irregularity (Ma et al., 2014), a difficulty in patients with one missing maxillary lateral incisor. When treating such cases with orthodontic SC, an aesthetic result is more difficult to achieve, since there is almost always a difference in size, color, and shape between the canine and the lateral incisor (Zachrisson et al., 2011). SC in such cases involves the risk of creating an asymmetry in the dentition, which can be un‐aesthetic. This can be avoided through grinding, whitening, porcelain veneers, or composite restorations (Zachrisson et al., 2011). In our study, the case rated as the one with the best overall aesthetics among the SC cases was a case with unilateral agenesis of the maxillary lateral incisor, where no restorations or whitening had been performed. This is an important finding and shows that there are situations where SC is a good treatment alternative even in cases with unilateral agenesis anteriorly in the maxilla.

We found that orthodontists rated the midline as better than the laypeople in both groups. This could be due to laypeople being more doubtful of the concept of the midline and therefore chose to place their mark in the middle of the VAS. The number of cases with bilateral aplasia of the maxillary lateral incisor and cases with unilateral aplasia differed between the IT group and the SC group. Despite this, the ratings of the midline did not differ between the IT group and the SC group when all assessors were included. Previous studies have found that orthodontists are less tolerant to discrepancies in the dental midline than laypeople (Pinho et al., 2007).

Robertsson et al. (2010) studied how different outcomes of treatment were perceived by professionals and laypeople after SC or tooth replacement. The treatment methods in the evaluated cases were orthodontic SC, conventional prosthodontics and implants. They found that professionals and laypeople were of a different opinion concerning the treatment outcome in cases with missing maxillary lateral incisors, where the professionals generally were more positive in their evaluations. This is partly in agreement with our study. In the present study, the orthodontists preferred the aesthetics of the dentition in its entirety in the cases treated with SC. Others have found that when the aesthetics have been evaluated by orthodontists, general practitioners in dentistry, and by laypeople, the aesthetics of natural teeth were rated as more pleasing than any type of prosthesis (Armbruster et al., 2004a). This statement does not correlate with our findings, since we found no difference in the aesthetic evaluation of cases treated with SC or IT when all three groups of assessors were included.

Patients treated due to missing one or two maxillary lateral incisors are likely to have equally attractive smiles as persons with a complete dentition (De‐Marchi et al., 2014). In studies by Armbruster et al. (2004a) and Schneider et al. (2016), it was concluded that the perception of aesthetics in the dentition can vary between laypeople and dental professionals, which is in concordance with our findings. Orthodontists in general are more susceptible to noticing deviations than general practitioners, and laypeople are least likely to notice a deviation in the aesthetics in the dentition (Armbruster et al., 2004b; Kokich et al., 1999; Pinho et al., 2007). These findings are not in correlation with the findings in the study by De‐Marchi et al. (2014), who found no difference between the aesthetic ratings of the dentition in cases treated due to missing maxillary laterals when comparing ratings of dentists and laypeople.

In the present study there are a few shortcomings. One of them is the presence of soft tissue in the pictures, which can influence the ratings concerning the dentition in its entirety. The use of color photographs of the lower third of the face has been widely used in previous studies (Brisman, 1980; De‐Marchi et al., 2014; Pinho et al., 2007) and photographs are considered to be a valid and reliable instrument in evaluating the aesthetic perception of a smile (Howells & Shaw, 1985). Perhaps one could criticize that the lips and the skin could interfere with the perception of the dentition. On the other hand, this is a more realistic situation for laypeople. We used printed copies in this study to eliminate the risk of varying color presentation on different electronic screens, which could have been a risk if the photographs had been sent to the assessors by email. Advising the assessors of what they were looking for could potentially bias them to look more closely at the potential issues affiliated with implants versus SC.

Another limitation is that this is a quantitative study relying on group values, which can conceal individual opinions.

The questionnaire was comprehensive and time‐consuming, which made retest evaluations almost impossible, and we chose to use a confirming question instead.

The aspect of time in this study should be seen as something both positive and negative. The negative aspect is that since the IT was performed, implant materials have been developed that allow clinicians to choose more aesthetic materials. The positive aspect is that we aim to give our patients a lifelong treatment and to do that we need to have long‐term follow‐ups.

## 5 CONCLUSION

The color of the gingiva adjacent to the implant‐supported crowns had a lower aesthetic rating than the SC group. Laypeople rated both treatments as equally good. The orthodontists had a slight preference for the aesthetics in the SC cases. The young laypeople were slightly more dissatisfied with the color of the tooth in the SC group compared to the older laypeople and the orthodontists.

## CONFLICT OF INTEREST

The authors declare that they have no conflict of interest.

## AUTHOR CONTRIBUTIONS

Cecilia Hedmo planned the research, collected and analyzed data, and wrote the paper. Rune Josefsson planned the research, collected and analyzed data, and wrote the paper. Eva Lindsten planned the research, analyzed data, and wrote the paper.

## Supporting information


**Appendix** **S1**: Supporting Information

## Data Availability

The data that support the findings of this study are available from the corresponding author upon request.
