# Supplementary material for: Laypeople and dental professionals' perception of the aesthetic outcome of two treatments for missing lateral incisors
Source: Clin Exp Dent Res. 2021 Oct 15;8(1):262–9. doi: 10.1002/cre2.504 (PMC8874116; doi:10.1002/cre2.504)
Supplement: Supplementary file 1 — Appendix S1: Supporting Information [file CRE2-8-262-s001.docx]

**Number: ____ Questionnaire**

Mark your assessment with one or two crosses on the line (one cross if it is one tooth to be assessed and two crosses if there are two teeth to be assessed).

*When two teeth are to be assessed, don´t forget to mark the cross with the number of the tooth to which the assessment applies, on question number three to five. Write 13, 12, 22 or 23 at the cross. Please see the attached example.*

**1. How pleases are you with the looks of the patients dentition?**

Very dissatisfied Very satisfied

l**____________________________________________________**l**­­­­**

**2. Do you think the dentition of the patient looks better or worse than people’s dentition in general?**

Much worse Much better

l**____________________________________________________**l**­­­­**

**3. How pleased are you with the shape of the marked tooth/teeth?**

Very dissatisfied Very satisfied

l**_________________________________________________________** l**­**

**4. How pleased are you with the colour of the marked tooth/teeth?**

Very dissatisfied Very satisfied

l**________________________________________________________** l**­­­­**

**5. How pleased are you with the colour of the gingiva adjacent to the marked tooth/teeth?**

Very dissatisfied Very satisfied

l**________________________________________________________**l**­­­­**

**6. How pleased are you with the midline in the upper jaw?**

Very dissatisfied Very satisfied

l**________________________________________________________** l

**7. In general, how pleased are you with the aesthetics with the dentition of the patient’s dentition?** (make a ring around the alternative you choose)

A. Yes, I´m very pleased.

B. I think it´s acceptable.

C. I don´t think it´s acceptable.

If you are not pleased with the aesthetics of the dentition, can you describe with your own words what you think is wrong?

____________________________________________________
